# Identification and Antibiotic Susceptibility Patterns of Clinical Blood Culture Isolates Not Identified by a Rapid Microarray Diagnostic System

**DOI:** 10.1128/spectrum.00175-21

**Published:** 2021-06-30

**Authors:** Jeffrey A. Freiberg, Connor R. Deri, Whitney J. Nesbitt, Romney M. Humphries, George E. Nelson

**Affiliations:** a Division of Infectious Diseases, Department of Medicine, Vanderbilt University Medical Center, Nashville, Tennessee, USA; b Department of Pharmacy, Vanderbilt University Medical Center, Nashville, Tennessee, USA; c Department of Pathology, Microbiology, and Immunology, Vanderbilt University Medical Center, Nashville, Tennessee, USA; University of Cincinnati

**Keywords:** antibiotic resistance, blood culture, diagnostics, Gram-negative bacteria, Gram-positive bacteria, molecular methods, Verigene

## Abstract

The use of molecular-based diagnostic testing, such as the Luminex Verigene system, to rapidly identify the most common bacterial isolates from blood cultures is an important tool that reduces the duration of inappropriate antibiotics and decreases mortality. However, 5 to 15% of microorganisms recovered from blood culture are unable to be identified by the Verigene Gram-negative (BC-GN) or Gram-positive (BC-GP) assays. In this retrospective, observational study, we evaluate the identities and antimicrobial susceptibility patterns of 229 isolates that were not identified by either the Verigene BC-GN or BC-GP assay. The results presented here suggest that important, clinically relevant information about antimicrobial susceptibility patterns can still be inferred even when isolates are not identified by Verigene. We also examined changes in antibiotic use for patients with “unidentified” Verigene results at our institution and found that this subgroup represents an opportunity to optimize empirical antibiotic therapy.

**IMPORTANCE** Rapid diagnostic testing to identify bloodstream pathogens has arisen as an important tool both to ensure adequate antimicrobial therapy is given early and to aid in antimicrobial stewardship by allowing for more rapid deescalation of inappropriate antimicrobial therapy. However, there is a paucity of data regarding the significance of isolates that are not able to be identified by rapid diagnostic testing. In this study, we report the identification to the species level and antimicrobial susceptibilities among isolates that were not identified by one such rapid diagnostic platform, the Verigene system. This study provides important insight into how a strong understanding of the strengths and limitations of a given rapid diagnostic platform, coupled with insight into local antibiotic susceptibility patterns, can allow for more nuanced and thoughtful empirical antibiotic selection.

## INTRODUCTION

In patients with bloodstream infections, early administration of appropriate antibiotics is important to decrease mortality, morbidity, and length of hospitalization (1–4). Therefore, it is not surprising that a major area of innovation has been the development of rapid diagnostic tests that assist with identification of bloodstream pathogens and markers of antimicrobial resistance hours to days earlier than is possible by traditional methods. Multiple technologies have been developed for this purpose, including *in situ* hybridization, DNA microarray, and DNA amplification (5).

The Verigene system (Luminex, Northbrook, IL) applies separate DNA microarrays to detect Gram-positive and Gram-negative blood culture isolates (BC-GP and BC-GN, respectively). Use of BC-GP and BC-GN has been shown to decrease time to bacterial identification for blood cultures by more than 24 h compared with traditional laboratory culture techniques (6–14). Multiple studies have shown that the use of Verigene not only significantly reduces the duration of inappropriate antibiotics and expedites the time to optimal antibiotic therapy (6, 11, 15–17) but also leads to decreased mortality, particularly when it is combined with guidance from an antimicrobial stewardship program (ASP) (12, 13, 18).

However, multiplexed rapid diagnostic tests for blood cultures are limited by the fact that they can only detect the specific pathogens targeted. In the case of the Verigene BC-GP, nine different Gram-positive organisms are identified at the species level and three are identified at the genus level (see Table S1 in the supplemental material). For Gram-negative organisms, the Verigene BC-GN microarray detects four different species and four different genera. Verigene can also detect three different resistance genes in Gram-positive organisms (*mecA*, *vanA*, and *vanB*) and six different resistance genes in Gram-negative organisms (the extended-spectrum beta-lactamase *bla*_CTX-M_ along with the *bla*_KPC_, *bla*_IMP_, *bla*_VIM_, *bla*_NDM_, and *bla*_OXA_ carbapenemase genes). The presence of a resistance mechanism is only reported when an organism is correctly identified, however. This combination of species and genera includes the most common bacteria isolated from the bloodstream (19–21), which allows the Verigene system to identify most clinical bloodstream isolates. This has been confirmed by multiple clinical studies in which Verigene correctly identifies isolates recovered from blood cultures to the genus or even species level greater than 90% of the time (8–10, 22–28). The Verigene system performs significantly better with monomicrobial cultures, however, with estimates of accuracy for polymicrobial cultures ranging from approximately 50 to 70% for Gram-negative organisms and 60 to 75% for Gram-positive organisms (8, 9, 14, 23, 28–31).

Previous studies have found approximately 5 to 8% of Gram-positive blood culture isolates and 5 to 15% of Gram-negative blood culture isolates are not identified by Verigene (7, 8, 12, 22, 25, 27). While a number of these isolates are species often thought to be contaminants in blood cultures (such as *Corynebacterium*, *Rothia*, *Micrococcus*, *Bacillus*, and *Gemella* species), a substantial number of true pathogens remain unidentified (*Morganella*, *Pasturella*, *Serratia*, *Stenotrophomonas*, and certain *Enterococcus* and Acinetobacter species, to name a few). In these cases, important clinical decision-making regarding antibiotic treatment must still occur despite the absence of early pathogen identification. There have been very few studies to date analyzing these isolates and the clinical decision-making surrounding isolates not identified by Verigene on a large scale. In this study, we analyzed all positive blood cultures over a 17-month period from a single institution’s microbiology laboratory for which the Verigene system was utilized, but no organism was identified. We further characterized these isolates based on their subsequent culture-based antimicrobial susceptibility testing (AST) results. In addition, we investigated the real-world changes in antibiotic usage associated with the release of a “not-identified” Verigene result.

## RESULTS

**FIG 1:**
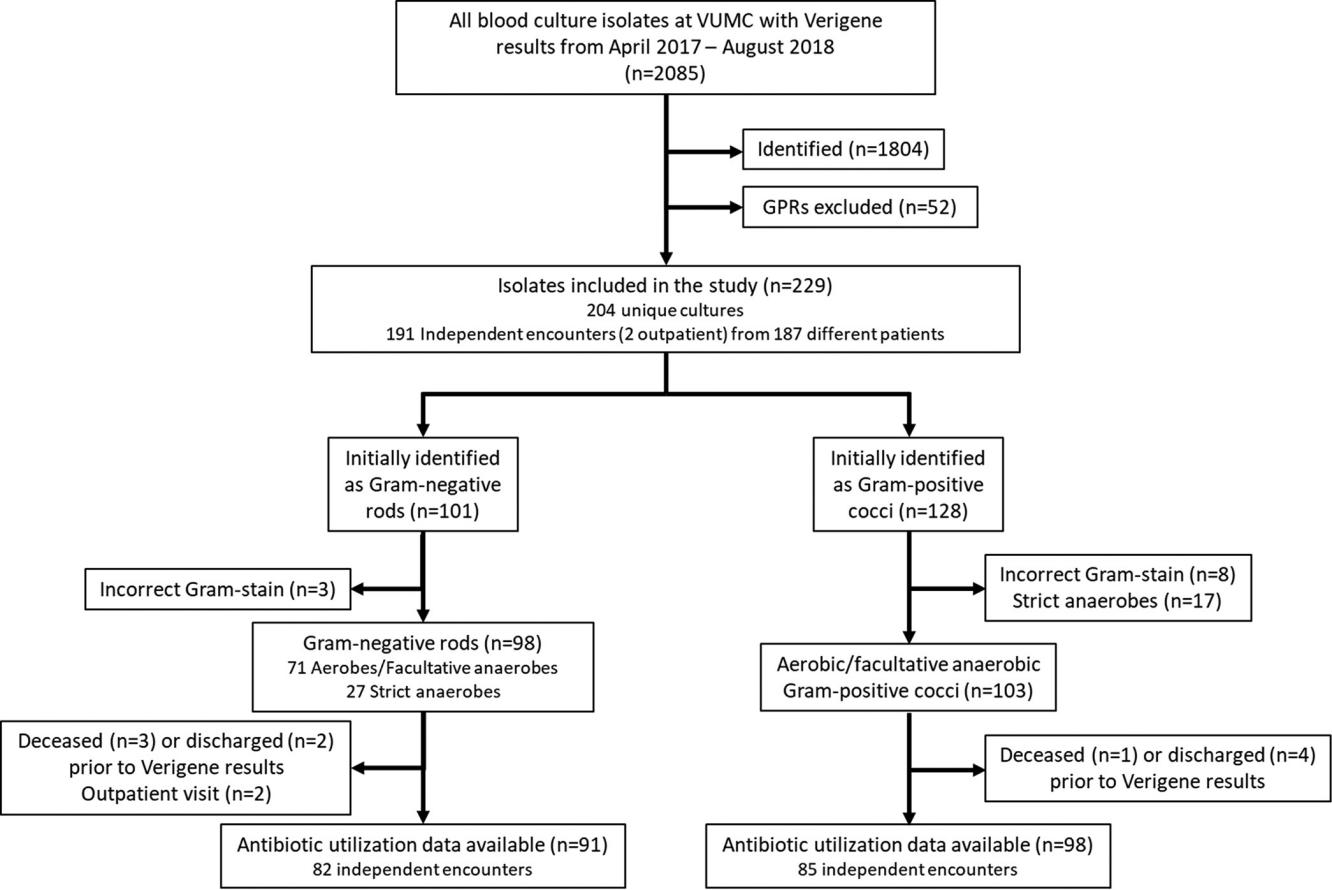
Flow diagram showing breakdown of isolates included in this study.

A total of 2,085 blood culture isolates that met inclusion criteria and for which Verigene results were available were recovered during the study period. The breakdown and distribution of these isolates are shown in Fig. 1. Of these 2,085 isolates, 52 were identified as Gram-positive rods (GPRs), leaving 2,033 isolates, of which 229 (11.3%) from a total of 204 cultures were not identified by Verigene. Nearly one-fifth of cultures with one or more unidentified microorganisms (40/204, 19.6%) were polymicrobial. The 204 cultures came from 191 separate encounters, as some patients had multiple cultures that could not be identified. The vast majority of these cultures were collected in an emergency department or inpatient setting, with only 2 cultures (0.9%) collected from outpatient clinics. Furthermore, as some patients had multiple hospital encounters, there were only 187 unique patients represented among the 191 separate encounters. The characteristics of the 189 distinct hospital encounters (outpatient encounters excluded) are shown in Table 1. Among the hospitalized patients included in this study, there was an in-hospital mortality of 10.0% (19/189) and a 30-day all-cause mortality of 14.6% (26/178). This is roughly similar, although slightly lower, to other published estimates of the mortality rates associated with bacteremia (12, 32–34).

**TABLE 1:** Demographics and clinical characteristics of patients ≥18 years of age for whom Verigene was unable to identify a pathogen^*a*^

Parameter	Value
Median age (IQR)	59 (43–69)
Median length of total hospital stay, days (IQR)	8 (5–15)
Percent requiring ICU stay	86 (45.5%)
Median length of ICU stay (IQR)	4.7 (1.9–8.0)
Percent of cultures collected in ICU (*n* = 204)	56 (27.3%)
In-hospital mortality	19 (10.0%)
30-day mortality (*n* = 178)	26 (14.6%)

a Represents data for the 189 distinct encounters for which complete discharge data were available. Thirty-day survival data were only available for 178 of the 189 encounters. IQR, interquartile range; ICU, intensive care unit.

The 229 microbial isolates not identified by Verigene encompassed 101 isolates that stained Gram negative (44.1%) and 128 isolates that stained Gram positive (55.8%) (Tables 2 and 3, respectively). Among the Gram-negative organisms not identified by Verigene, the vast majority were aerobic Gram-negative rods (GNRs), with *Serratia* the most common Gram-negative genus not identified by Verigene (22 isolates, 21.8% of all Gram-negative isolates), followed by *Bacteroides* with 19 isolates (18.8% of all Gram-negative isolates). Also notable was the fact that there were 10 isolates (9.9%) of Stenotrophomonas maltophilia. Three isolates that were initially identified as GNRs on Gram stain ended up being determined at the species level as GPRs.

**TABLE 2:** Gram-negative organisms not identified by Verigene^*a*^

Genus	No. of isolates
Aerobic/facultative anaerobic Gram-negative rods	71
*Achromobacter*	4
*Aeromonas*	1
*Burkholderia*	2
Campylobacter	2
*Chryseobacterium*	1
*Edwardsiella*	1
**Escherichia coli**	3
Haemophilus	3
*Hafnia*	1
**Klebsiella pneumoniae**	5
*Morganella*	1
*Pantoea*	4
*Pasteurella*	3
*Providencia*	2
Pseudomonas (non-*aeruginosa*)	2
*Rhizobium*	1
*Roseomonas*	1
Salmonella	2
*Serratia*	22
S. marcescens	20
Other *Serratia* spp.	2
*Stenotrophomonas*	10
Strictly anaerobic Gram-negative rods	27
*Bacteroides*	19
*Fusobacterium*	1
*Leptotrichia*	2
*Prevotella*	2
*Tissierella*	1
Other anaerobic Gram-negative rod	2
Aerobic Gram-positive rods^*b*^	3
*Bacillus*	1
*Lactobacillus*	2

a *N* = 101. Boldface text indicates genus/species that should be identified via Verigene.

b Initially identified as Gram negative on Gram stain.

**TABLE 3:** Gram-positive organisms not identified by Verigene^*a*^

Genus	No. of isolates
Aerobic Gram-positive rods^*b*^	2
*Lactobacillus*	1
Other Gram-positive rods	1
Aerobic/facultative anaerobic Gram-positive cocci	103
*Aerococcus*	3
*Dermacoccus*	1
*Enterococcus*	9
**E. faecalis**	2
**E. faecium**	2
Other *Enterococcus* spp.	5
*Gemella*	6
*Leuconostoc*	1
*Micrococcus*	18
*Rothia*	5
**Staphylococcus**	25
**S. aureus**	3
**S. epidermidis**	10
Other coagulase-negative Staphylococcus	12
**Streptococcus**	25
**S. pyogenes**	1
**S. agalactiae**	1
S. dysgalactiae	1
S. bovis	1
***S. anginosus* group**	5
viridans Streptococcus, not *anginosus* group	16
Streptococcus-nutritionally deficient	9
Other Gram-positive cocci	1
Anaerobic Gram-positive cocci	17
*Anaerococcus*	5
*Finegoldia*	3
*Parvimonas*	3
*Peptoniphilus*	2
*Peptostreptococcus*	4
Yeast^*b*^	1
*Candida*	1
Aerobic/facultative anaerobic Gram-negative rods^*b*^	2
**Acinetobacter baumannii**	2
Aerobic Gram-negative cocci^*b*^	1
*Neisseria*	1
Anaerobic Gram-negative cocci^*b*^	2
*Veillonella*	2

a *N* = 128. Boldface text indicates genus/species that should be identified via Verigene.

b Initially identified as Gram-positive cocci on Gram stain.

Among the 128 Gram-positive isolates, most were streptococci or staphylococci, with each genus representing 19.5% of all Gram-positive isolates. This included three Staphylococcus aureus isolates (2.3%). One isolate was initially identified as a Gram-positive organism but was ultimately determined to be a polymicrobial culture with Candida tropicalis in addition to a Gram-positive cocci (GPC) upon culture growth. Additionally, five cultures that were identified as GPCs on initial Gram stain resulted as Acinetobacter, *Veillonella*, or *Neisseria* species.

Slightly more than one-fourth (28.4%; 65/229) of the isolates were expected to be identified by Verigene. As shown in Table 4, over one-third of these isolates were from polymicrobial cultures. However, the overall accuracy of the Verigene was relatively high. In total, 1,879 blood culture isolates were organisms targeted by Verigene, and these were identified correctly more than 96% of the time.

**TABLE 4:** Isolates that should have been identified by Verigene

Bacterium	No. (%) of isolates not identified/total number	No. (%) isolated from polymicrobial cultures^*b*^
Gram positive		
E. faecalis	2/68 (3)	0/2 (0)
E. faecium	2/34 (6)	0/2 (0)
*Listeria* spp.	0/3 (0)	NA
S. aureus	3/387 (1)	0/3 (0)
S. epidermidis	10/451 (2)	7/10 (70)
*S. lugdunensis*	0/3 (0)	NA
Other Staphylococcus spp.	12/305 (4)	5/12 (42)
S. pyogenes	1/10 (10)	0/1 (0)
S. agalactiae	1/22 (5)	0/1 (0)
S. pneumoniae	0/13 (0)	NA
*S. anginosus* group	6/27 (22)	1/6 (17)
Other Streptococcus spp.	18/113 (16)	8/18 (44)
Total	55/1445 (4)	21/55 (38)
Gram negative		
Acinetobacter spp.^*a*^	2/21 (10)	0/2 (0)
*Citrobacter* spp.	0/8 (0)	NA
Enterobacter spp.	0/38 (0)	NA
E. coli	3/217 (1)	2/3 (67)
K. oxytoca	0/17 (0)	NA
K. pneumoniae	5/77 (6)	0/5 (0)
Proteus spp.	0/12 (0)	NA
P. aeruginosa	0/12 (0)	NA
Total	10/434 (2)	2/10 (20)

a Both missed Acinetobacter isolates were initially identified as Gram-positive cocci on Gram stain.

b NA, not applicable.

**FIG 2:**
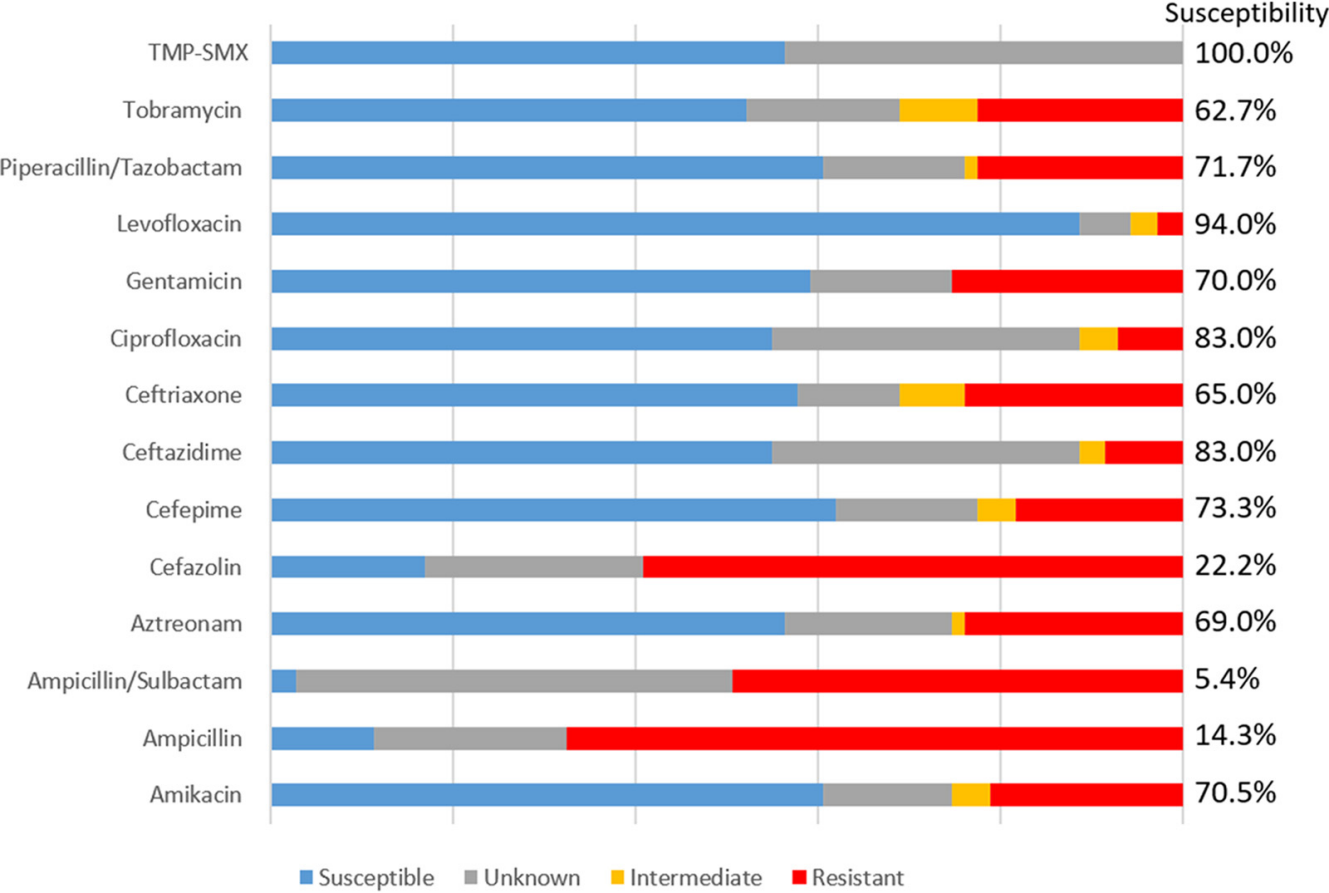
Resistance patterns for aerobic and facultative anaerobic GNRs blood culture isolates not identified by Verigene. Composite data from antibiotic susceptibility testing along with intrinsic resistance shown. Isolates were presumed to be susceptible if susceptibility testing was not done on that isolate but was done on a separate isolate of the same species from the same patient during the same admission. Percent susceptibility displayed reflects the percentage of susceptible isolates among all isolates with either antimicrobial susceptibility testing data or known intrinsic resistance. Percent susceptibility is not displayed in cases where more than 50% of isolates were not tested for susceptibility to a given antibiotic. *N* = 71.

**FIG 3:**
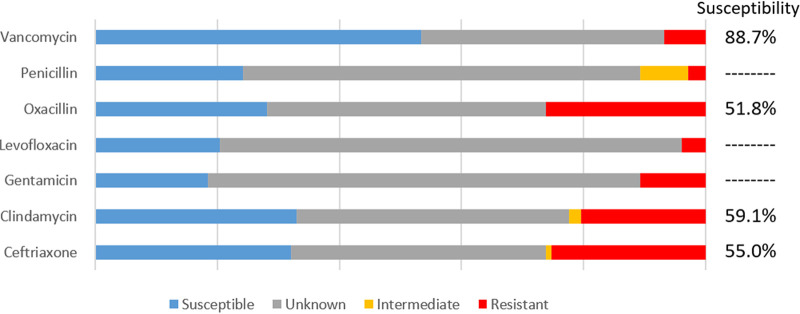
Resistance patterns for aerobic GPCs blood culture isolates not identified by Verigene. Composite data from antibiotic susceptibility testing along with intrinsic resistance shown. Isolates were presumed to be susceptible if susceptibility testing was not done on that isolate but was done on a separate isolate of the same species from the same patient during the same admission. Percent susceptibility displayed reflects the percentage of susceptible isolates among all isolates with either antimicrobial susceptibility testing data or known intrinsic resistance. Percent susceptibility is not displayed in cases where more than 50% of isolates were not tested for susceptibility to a given antibiotic. *N* = 103.

Among the isolates not identified by Verigene, antibiograms were only constructed for aerobic/facultative anaerobic GNRs along with aerobic/facultative anaerobic GPCs, as most isolates from other groups of bacteria (anaerobes, GPRs, and Gram-negative cocci) were not routinely subjected to AST at our institution. The resistance patterns for the GNRs and GPCs included in this study are shown in Fig. 2 and 3, respectively. Susceptibility patterns categorized by location are also shown, both for isolates collected in an intensive care unit (ICU) (see Fig. S1 and S2 in the supplemental material) and a non-ICU setting (Fig. S3 and S4).

**FIG 4:**
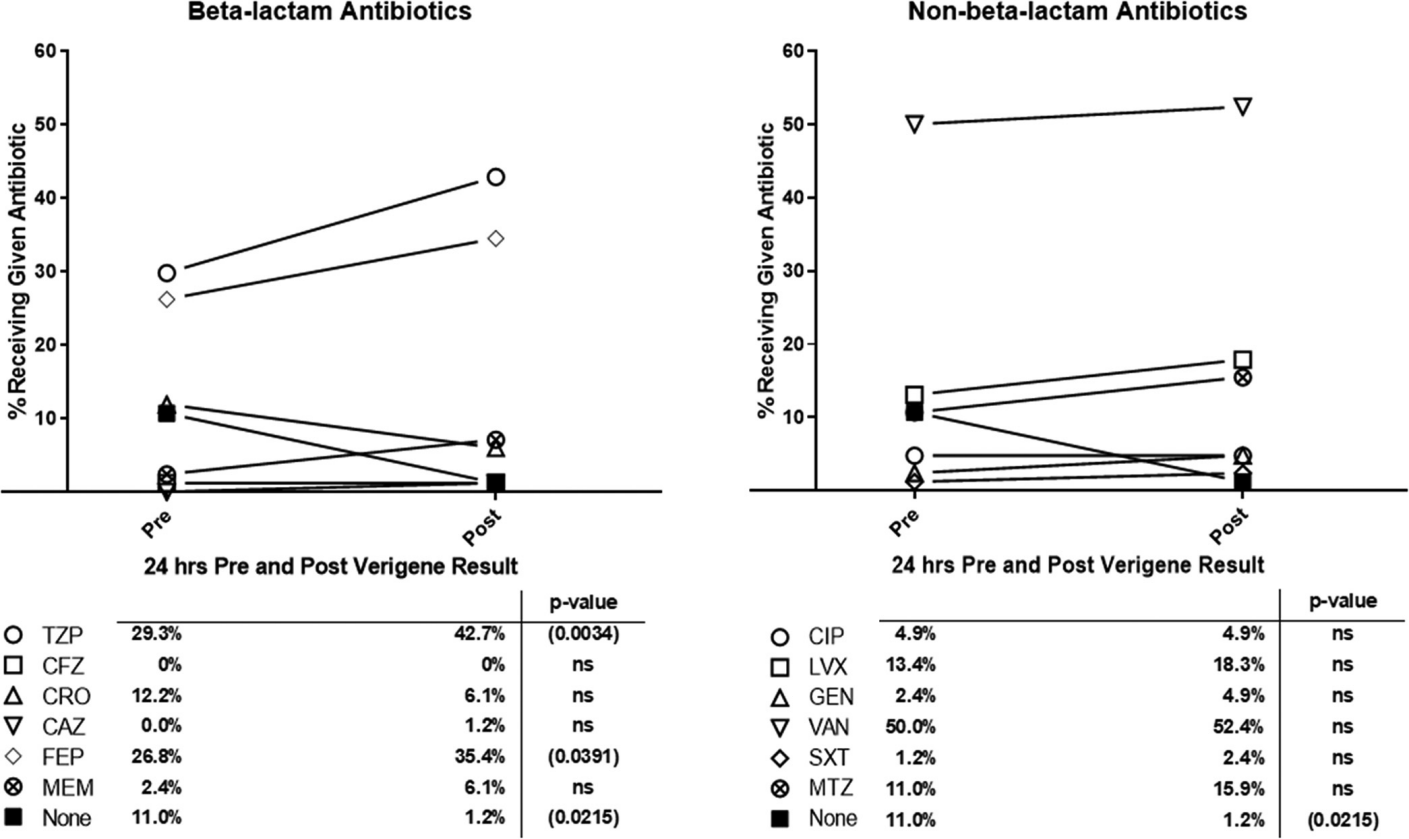
Change in antibiotic utilization after detection of GNRs not identified by Verigene. Shown is the percentage of patients receiving a given antibiotic who had GNRs isolated from a blood culture but not identified by Verigene (for whom antibiotic administration data were available). Pre represents any use of the given antibiotic in the 24-h period prior to the Gram stain and Verigene result, while post represents any use of the antibiotic in the 24 h following the Gram stain and Verigene result. *N* = 82. TZP, piperacillin-tazobactam; CFZ, cefazolin; CRO, ceftriaxone; CAZ, ceftazidime; FEP, cefepime; MEM, meropenem; CIP, ciprofloxacin; LVX, levofloxacin; GEN, gentamicin; VAN, vancomycin; DAP, daptomycin; SXT, sulfamethoxazole-trimethoprim; MTZ, metronidazole.

**FIG 5:**
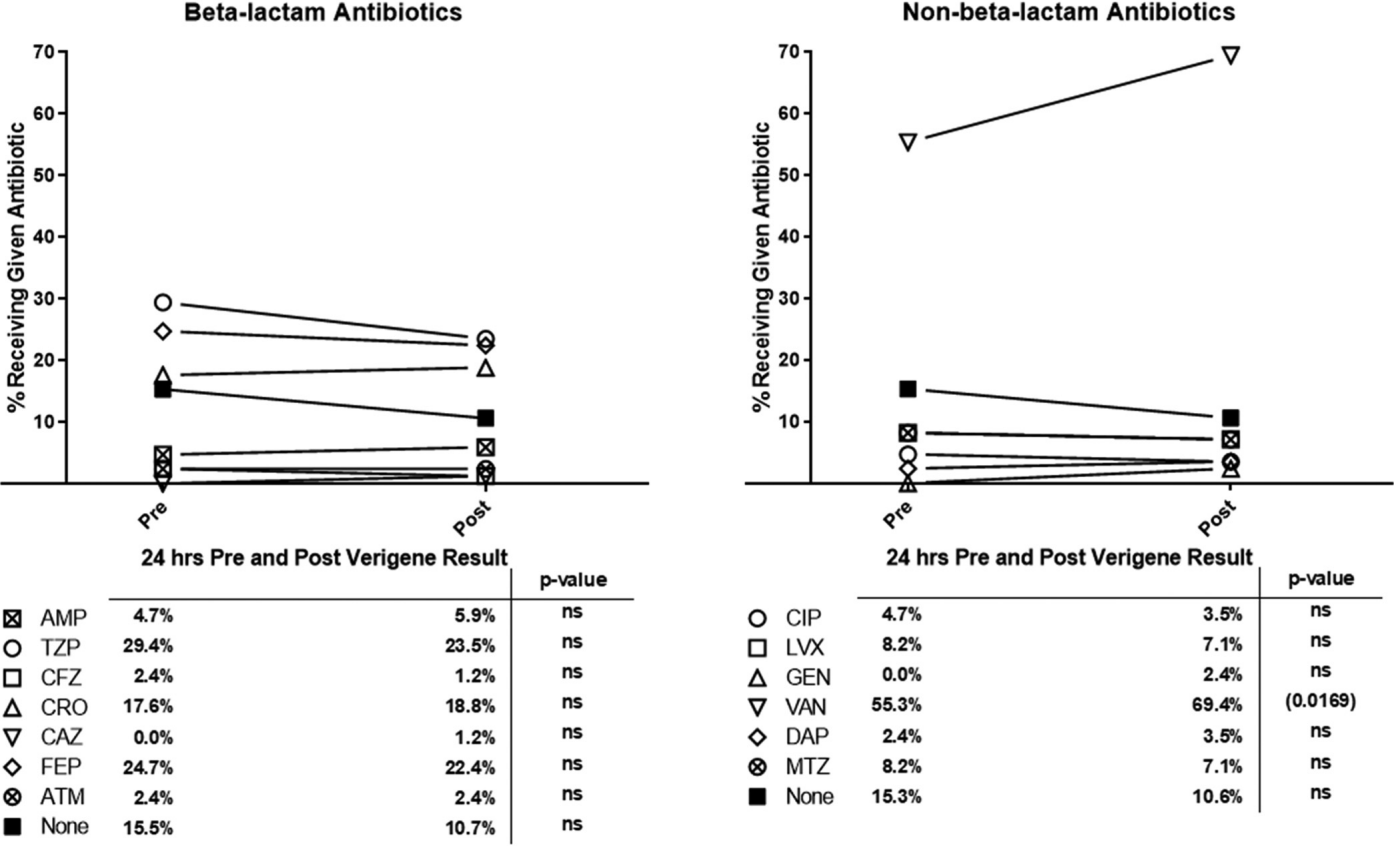
Change in antibiotic utilization after detection of aerobic GPCs not identified by Verigene. Shown here is the percentage of patients receiving a given antibiotic who had aerobic GPCs isolated from a blood culture but not identified by Verigene (for whom antibiotic administration data were available). Pre represents any use of the given antibiotic in the 24 h prior to the Gram stain and Verigene result, while post represents any use of the antibiotic in the 24 h following the Gram stain and Verigene result. *N* = 85. AMP, ampicillin; TZP, piperacillin, tazobactam; CFZ, cefazolin; CRO, ceftriaxone; CAZ, ceftazidime; FEP, cefepime; ATM, aztreonam; CIP, ciprofloxacin; LVX, levofloxacin; GEN, gentamicin; VAN, vancomycin; DAP, daptomycin; MTZ, metronidazole.

Antibiotic use in the 24 h prior to and the 24 h after Verigene results became available in the electronic medical record for GNRs and GPCs is displayed in Fig. 4 and 5, respectively. Unsurprisingly, nearly all patients (98.8%; 81/82) received antibiotics in the 24-h period after unidentified GNRs were isolated from a blood culture (compared to 89% prior to a positive blood culture). This included a significant increase in use of beta-lactam antibiotics (69.5% versus 86.6%; *P* < 0.001), which was driven by a 13.4% increase in piperacillin-tazobactam use (29.3% versus 42.7%; *P* = 0.003). Notably, the use of several antibiotics with antipseudomonal coverage (piperacillin-tazobactam, cefepime, levofloxacin, and meropenem) increased despite there not being any Pseudomonas aeruginosa among the isolates included in this study. There was also a corresponding decrease in the use of ceftriaxone despite there being similar levels of resistance to ceftriaxone and piperacillin-tazobactam (the most commonly used antibiotic after the identification of GNRs in this study), particularly among isolates collected in an ICU (Fig. S1 and S2).

Interestingly, the growth of GPCs that could not be identified by Verigene did not result in the same degree of antibiotic usage. Over 10% of patients did not receive any antibiotics in the 24-h period after the detection of GPCs and the release of Verigene results. The rates of beta-lactam usage did not increase (going from 72.9% to 71.8%). Instead, there was a significant increase in vancomycin use, going from 55.3% to 69.4% (*P* = 0.017) when GPCs that could not be identified by Verigene were isolated.

## DISCUSSION

In this study, we characterized the blood culture isolates not identified by Verigene using real-world clinical microbiology laboratory results. We also provide susceptibility patterns for these isolates that may inform more targeted empirical antibiotic treatment. Verigene performed well when it came to identifying the isolates it is designed to target, with only a small fraction of the isolates that should have been identified being missed, and many of these were cases where the bacteria were found in polymicrobial cultures, a previously recognized weakness for Verigene (8, 9, 14, 23, 28–31).

Among the isolates not expected to be identified by Verigene, Serratia marcescens represented a large proportion of the unidentified gram negatives (Table 2). Fortunately, this species is targeted in newer generations of the Verigene assay (R. M. Humphries, personal communication with Luminex) in addition to being currently available in the research-use-only (RUO) version of the BC-GN. Inclusion of *Serratia* in the Verigene assay would have reduced the number of Gram-negative isolates not identified in this study by greater than one-fifth (21.8%, 22/101). The total percentage of isolates not identified by the Verigene assay would have decreased from 11.0% to 9.9% (207/2,085) if all 22 of the *Serratia* isolates were identified. Notably, there were 5 isolates reported to be Klebsiella pneumoniae that were not identified by the Verigene assay. While this suggests a higher degree of inaccuracy among Klebsiella pneumoniae isolates, it may also reflect difficulties in correctly identifying K. pneumoniae by traditional microbiologic methods, as were used in this study. This was reflected in several studies that found, once genetic sequencing techniques were applied, many isolates initially classified as K. pneumoniae yet not identified by Verigene were, in fact, isolates of Klebsiella variicola (29, 30).

For Gram-positive organisms, staphylococci and streptococci were the most common organisms not identified by Verigene. This is despite there being genus-level targets for both Streptococcus and Staphylococcus included in the microarray, which is indicative of the inherent difficulty in making a highly sensitive probe against such large and variable genera. However, it is also possible that reliance on traditional microbiological methods, as was the case during the study period, resulted in some misidentifications.

Among the GNRs isolated in this study, as shown in Fig. 2, there was a very high degree of susceptibility to levofloxacin both in terms of absolute numbers of sensitive isolates and percent susceptibility (94% among isolates tested). A similar antibiogram was seen for isolates collected in an ICU (see Fig. S1 in the supplemental material). Overall, the isolates included in this study were less susceptible to antibiotics than the overall GNR antibiogram (for all isolates, whether or not they were identified by the Verigene; data not shown) for this time period. For most antibiotics, the percentage of susceptible isolates among GNRs included in this study was 10 to 20% lower than what was seen in the general GNR antibiogram. The notable exceptions were the fluoroquinolones (ciprofloxacin and levofloxacin) and trimethoprim-sulfamethoxazole, as all three of these antibiotics had much higher susceptibility rates among the isolates in this study than the general GNR antibiogram. Although S. marcescens was the most common GNR isolate in this study, S. marcescens isolates during the study period actually demonstrated more susceptibility than the overall GNR antibiogram, suggesting they were not the factor that was driving the higher rates of resistance seen in this study.

A much smaller percentage of Gram-positive isolates had susceptibility testing done. This reflects that many of these isolates are species that are not routinely subjected to AST. While this makes it harder to generalize about antimicrobial susceptibility, vancomycin was clearly the superior choice for empirical Gram-positive antibiotic coverage. Not only was vancomycin the most frequently tested, it also was the antibiotic with the greatest degree of susceptibility (88.7%). Vancomycin resistance was noted only among some of the *Enterococcus* isolates, the majority of which still retained susceptibility to penicillins. This contrasts with the overall GPC antibiogram, however, which showed a greater than 98% susceptibility to vancomycin (data not shown).

There were several limitations to this study. Most notably, as this study was done at a single site, institutional practices and local antibiotic susceptibility patterns undoubtedly had a strong influence on both the antibiograms reflected in Fig. 2 and 3 and the empirical antibiotic decision-making reflected in Fig. 4 and 5. Since all antibiotic use occurs in the context of the local microbiologic milieu, any study of antibiotic susceptibility patterns is inherently limited in its generalizability. This potentially limits the clinical utility of pooled data gathered from a multisite study. However, our study suggests there is potential for ASPs to utilize their own institutional-level data to construct similar antibiograms for unidentified isolates locally. It is possible that this information could be used to inform empirical therapy while awaiting traditional susceptibility results. However, the breakdown of the identities of the isolates shown in Tables 2 and 3 that were not originally identified by the Verigene is likely more broadly applicable. The success rate in this study for identifying organisms that the Verigene would be expected to recognize (97.7% for Gram-negative organisms and 95.6% for Gram-positive organisms) aligns with other studies (8–10, 14, 22–31).

Additionally, as this was a retrospective, observational study, it is difficult to discern what role the Verigene results played in clinical decision-making surrounding antibiotic selection. Our study design, unfortunately, did not allow for us to control for patient’s clinical statuses when assessing empirical antibiotic choices. Although Fig. 4 and 5 give some window into how antibiotic selection changed due to the presence of an unidentified blood culture isolate, it does not acknowledge the role that prior blood cultures or cultures from other sites may have played in antibiotic selection. It is also possible that many patients were clinically stable or improving on the antibiotic regimen initially selected, so no changes were made. As this was a descriptive study, no ASP guidance was provided based on the Verigene results outside the routine ASP activity at our institution. Implementing an ASP intervention based on the Verigene results would likely lead to a more pronounced difference in the pre- and postantibiotic usage.

There were some clear trends in antibiotic usage that coincided with the timing of the Verigene results. Antibiotic usage was greater after GNRs were isolated from blood cultures compared to when GPCs were isolated. It is possible that this difference in empirical antibiotic decision-making reflects differences in the clinical context between patients who develop Gram-negative bacteremia versus Gram-positive bacteremia. It is also conceivable that this reflects the fact that clinicians are more likely to attribute GPCs in a blood culture that are unidentified to a skin contaminant than they are for unidentified GNRs. Given the challenges inherent in trying to classify bacteria as potential contaminants, we included all isolates tested on the Verigene system in our study and did not remove any isolates, even ones that were likely to have been contaminants.

It is also possible that the presence of GPRs in some blood cultures affected the antibiotic usage patterns seen. GPRs made up a very small portion (2.5%, 52/2085) of the samples run on the Verigene system. This is reflective of the fact that since GPRs are often contaminants in cultures and only one genus of GPRs (*Listeria*) is targeted by Verigene, they are not routinely analyzed. A lack of understanding of the role GPRs are playing in antibiotic selection is a potential limitation of this study.

For unidentified GNRs, there was frequent broadening of antibiotics to cover resistant GNRs and Pseudomonas, despite a lack of microbiological evidence to justify this approach. For GPCs, while vancomycin use increased, it remained less than 70% despite it being a good empirical choice given the high level of susceptibility among the isolates in this study. Overall, the results from this study suggest there are opportunities for providing additional antimicrobial guidance, even when rapid diagnostic testing cannot provide a microbial identity.

The mortality rates among patients included in this study are very similar to the mortality rates seen when looking at composite estimates of all bloodstream infections, regardless of organism (19, 20, 34). This reinforces the fact that even when an organism cannot be identified by rapid diagnostic testing, bacteremia still demands careful consideration given its high associated morbidity and mortality. As this study demonstrates, there is still a great deal of useful clinical information to be discerned from rapid microbiological diagnostic testing, even in the absence of a clear result. A strong understanding of the strengths and limitations of a given rapid diagnostic platform, such as the Verigene system, coupled with insight into local antibiotic susceptibility patterns can allow for more nuanced and thoughtful empirical antibiotic selection.

## MATERIALS AND METHODS

### Study design.

This retrospective study analyzed the identities of blood cultures tested using the Verigene BC-GP and BC-GN panels between April 2017 and August 2018. All positive blood culture reports from patients 18 years or older with Verigene test(s) performed by the Vanderbilt University Medical Center (VUMC) clinical microbiology laboratory were manually reviewed, including blood cultures drawn in the emergency department, the hospital wards, and outpatient settings. Records for which no organism was identified by Verigene, or for which multiple organisms were present and at least one was not identified by Verigene, were included for further analysis. For patients with multiple hospital encounters or multiple isolates not identified during the same encounter, all isolates subjected to Verigene testing were included for further review.

### Data collection.

For the positive blood cultures with no organism identified by Verigene, the following data were extracted from the patient’s medical record: age at time of culture, time of culture, time of Verigene result, hospital length of stay, whether they were in an ICU at the time the culture was obtained, ICU length of stay (if applicable), mortality prior to discharge, and 30-day mortality. The use of antimicrobials 2 days prior to the time of blood culture collection and up to 7 days after blood culture results was recorded. If patients were not hospitalized at the time at which Verigene results became available (whether due to discharge or death), they were not including in further analysis regarding changes in antimicrobial usage. Data were entered into secure REDcap forms and analyzed in Microsoft Excel and GraphPad Prism. This study was approved by the VUMC institutional review board.

### Blood culture analysis.

Blood cultures were performed using a Bactec FX (BD, Sparks MD) and Bactec plus aerobic and lytic anaerobic medium. Blood cultures were incubated a total of 5 days prior to being discarded as negative. Gram stain-directed BC-GP or BC-GN testing was available on a continuous basis and was performed at the time of the first positive blood culture with a unique Gram stain, per patient per hospital encounter. If the patient had a previous positive blood culture within 3 days, no molecular testing was performed, assuming the current Gram stain was consistent with the previous culture result. Cultures with Gram-negative rods (GNRs) were tested by BC-GN, and cocci (GPCs) were tested by the BC-GP. If both GNRs and GPCs were seen, both BC-GN and BC-GP panels were tested. Select Gram-positive rods (GPRs) were tested by BC-GP; however, as GPRs are not routinely tested by the Verigene at VUMC, they were excluded from further analysis. Results were reported to the medical record; if no target was detected, this too was reported in the electronic medical record, with reference to the VUMC antibiotic stewardship website for information on the test panels, and targets. All positive blood cultures were subcultured to blood, chocolate, and/or MacConkey agar plates following laboratory protocols, upon completion of the Gram stain. Colonial growth was identified using the Phoenix (BD) ID cards, API strips (bioMérieux, Durham NC), and spot biochemicals. If no colonial growth was evident at 24 h, or if Gram stain was suggestive of an anaerobic organism, blood cultures were subcultured to Brucella blood agar and incubated anaerobically for 48 h; anaerobic growth was identified by using RapidID ANA II (ThermoFisher, Lenexa, KS). If an identification was not possible, isolates were referred to the Tennessee Department of Health State Laboratory for identification. For each organism, targeted by the Verigene assay, the total number of isolates during the study period was determined by adding the number of isolates of a given organism identified correctly by the Verigene assay to the number of unidentified isolates of that organism. Isolates were considered to be from a polymicrobial culture if there were any other organisms present in the culture, regardless of whether the other organisms were identified by Verigene or not.

### Antimicrobial susceptibility testing.

AST results recorded in the medical record were used for this analysis. Susceptibility testing was performed using the Phoenix system (BD) PMIC 304 and NMIC 106 panels for Gram-positive and Gram-negative organisms, respectively. Information regarding which antibiotics are tested in these panels is included in Table S2 in the supplemental material, along with information on which species did not have routine antimicrobial susceptibility testing performed. Susceptibility testing for anaerobes, when performed, was performed by ARUP reference laboratories. Bacterial species with known intrinsic resistance to specific antimicrobial agents/classes were counted as resistant for those antibiotics, regardless of whether additional resistance testing was done (35).

### Statistical analysis.

Changes in antibiotic use pre- and post-Verigene results were analyzed using a two-tailed binomial test. All statistical analysis was done using GraphPad Prism v9.0 (GraphPad Software). A *P* value of <0.05 was considered significant.

## References

[1] Ibrahim EH, Sherman G, Ward S, Fraser VJ, Kollef MH. 2000. The influence of inadequate antimicrobial treatment of bloodstream infections on patient outcomes in the ICU setting. Chest 118:146–155. doi:10.1378/chest.118.1.146. 10893372

[2] Bauer KA, West JE, Balada-Llasat JM, Pancholi P, Stevenson KB, Goff DA. 2010. An antimicrobial stewardship program's impact with rapid polymerase chain reaction methicillin-resistant *Staphylococcus aureus*/*S. aureus* blood culture test in patients with *S. aureus* bacteremia. Clin Infect Dis 51:1074–1080. doi:10.1086/656623. 20879856

[3] Kumar A, Roberts D, Wood KE, Light B, Parrillo JE, Sharma S, Suppes R, Feinstein D, Zanotti S, Taiberg L, Gurka D, Kumar A, Cheang M. 2006. Duration of hypotension before initiation of effective antimicrobial therapy is the critical determinant of survival in human septic shock. Crit Care Med 34:1589–1596. doi:10.1097/01.CCM.0000217961.75225.E9. 16625125

[4] Leibovici L, Shraga I, Drucker M, Konigsberger H, Samra Z, Pitlik SD, 1998. The benefit of appropriate empirical antibiotic treatment in patients with bloodstream infection. J Intern Med 244:379–386. doi:10.1046/j.1365-2796.1998.00379.x. 9845853

[5] Peker N, Couto N, Sinha B, Rossen JW. 2018. Diagnosis of bloodstream infections from positive blood cultures and directly from blood samples: recent developments in molecular approaches. Clin Microbiol Infect 24:944–955. doi:10.1016/j.cmi.2018.05.007. 29787889

[6] Belknap A, Grosser DS, Hale DA, Lang BJ, Colley P, Benavides R, Dhiman N. 2017. Clinical uptake of antimicrobial stewardship recommendations following Nanosphere Verigene blood culture gram-negative reporting. Proc (Bayl Univ Med Cent) 30:395–399. doi:10.1080/08998280.2017.11930204. 28966443 PMC5595373

[7] Hill JT, Tran KD, Barton KL, Labreche MJ, Sharp SE. 2014. Evaluation of the nanosphere Verigene BC-GN assay for direct identification of gram-negative bacilli and antibiotic resistance markers from positive blood cultures and potential impact for more-rapid antibiotic interventions. J Clin Microbiol 52:3805–3807. doi:10.1128/JCM.01537-14. 25122857 PMC4187775

[8] Buchan BW, Ginocchio CC, Manii R, Cavagnolo R, Pancholi P, Swyers L, Thomson RB, Jr, Anderson C, Kaul K, Ledeboer NA. 2013. Multiplex identification of gram-positive bacteria and resistance determinants directly from positive blood culture broths: evaluation of an automated microarray-based nucleic acid test. PLoS Med 10:e1001478. doi:10.1371/journal.pmed.1001478. 23843749 PMC3699453

[9] Wojewoda CM, Sercia L, Navas M, Tuohy M, Wilson D, Hall GS, Procop GW, Richter SS. 2013. Evaluation of the Verigene gram-positive blood culture nucleic acid test for rapid detection of bacteria and resistance determinants. J Clin Microbiol 51:2072–2076. doi:10.1128/JCM.00831-13. 23596240 PMC3697701

[10] Mestas J, Polanco CM, Felsenstein S, Dien Bard J. 2014. Performance of the Verigene gram-positive blood culture assay for direct detection of gram-positive organisms and resistance markers in a pediatric hospital. J Clin Microbiol 52:283–287. doi:10.1128/JCM.02322-13. 24131696 PMC3911431

[11] Hayakawa K, Mezaki K, Kobayakawa M, Yamamoto K, Mutoh Y, Tsuboi M, Hasimoto T, Nagamatsu M, Kutsuna S, Takeshita N, Katanami Y, Ishikane M, Ohmagari N. 2017. Impact of rapid identification of positive blood cultures using the Verigene system on antibiotic prescriptions: a prospective study of community-onset bacteremia in a tertiary hospital in Japan. PLoS One 12:e0181548. doi:10.1371/journal.pone.0181548. 28742143 PMC5524366

[12] Walker T, Dumadag S, Lee CJ, Lee SH, Bender JM, Cupo Abbott J, She RC. 2016. Clinical impact of laboratory implementation of Verigene BC-GN microarray-based assay for detection of gram-negative bacteria in positive blood cultures. J Clin Microbiol 54:1789–1796. doi:10.1128/JCM.00376-16. 27098961 PMC4922078

[13] Claeys KC, Heil EL, Hitchcock S, Johnson JK, Leekha S. 2020. Management of gram-negative bloodstream infections in the era of rapid diagnostic testing: impact with and without antibiotic stewardship. Open Forum Infect Dis 7:ofaa427. doi:10.1093/ofid/ofaa427. 33134414 PMC7585329

[14] Dodémont M, De Mendonça R, Nonhoff C, Roisin S, Denis O. 2014. Performance of the Verigene gram-negative blood culture assay for rapid detection of bacteria and resistance determinants. J Clin Microbiol 52:3085–3087. doi:10.1128/JCM.01099-14. 24899026 PMC4136123

[15] Bork JT, Leekha S, Heil EL, Zhao L, Badamas R, Johnson JK. 2015. Rapid testing using the Verigene gram-negative blood culture nucleic acid test in combination with antimicrobial stewardship intervention against gram-negative bacteremia. Antimicrob Agents Chemother 59:1588–1595. doi:10.1128/AAC.04259-14. 25547353 PMC4325795

[16] Cwengros LN, Mynatt RP, Timbrook TT, Mitchell R, Salimnia H, Lephart P, Pogue JM. 2020. Minimizing time to optimal antimicrobial therapy for Enterobacteriaceae bloodstream infections: a retrospective, hypothetical application of predictive scoring tools vs rapid diagnostics tests. Open Forum Infect Dis 7:ofaa278. doi:10.1093/ofid/ofaa278. 32875001 PMC7452369

[17] Beal SG, Thomas C, Dhiman N, Nguyen D, Qin H, Hawkins JM, Dekmezian M, Benavides R, Njoku J. 2015. Antibiotic utilization improvement with the Nanosphere Verigene gram-positive blood culture assay. Proc (Bayl Univ Med Cent) 28:139–143. doi:10.1080/08998280.2015.11929214. 25829639 PMC4365105

[18] Rivard KR, Athans V, Lam SW, Gordon SM, Procop GW, Richter SS, Neuner E. 2017. Impact of antimicrobial stewardship and rapid microarray testing on patients with gram-negative bacteremia. Eur J Clin Microbiol Infect Dis 36:1879–1887. doi:10.1007/s10096-017-3008-6. 28534213

[19] Laupland KB, Kibsey PC, Gregson DB, Galbraith JC. 2013. Population-based laboratory assessment of the burden of community-onset bloodstream infection in Victoria, Canada. Epidemiol Infect 141:174–180. doi:10.1017/S0950268812000428. 22417845 PMC9152045

[20] Kollef MH, Zilberberg MD, Shorr AF, Vo L, Schein J, Micek ST, Kim M. 2011. Epidemiology, microbiology and outcomes of healthcare-associated and community-acquired bacteremia: a multicenter cohort study. J Infect 62:130–135. doi:10.1016/j.jinf.2010.12.009. 21195110

[21] Laupland KB. 2013. Incidence of bloodstream infection: a review of population-based studies. Clin Microbiol Infect 19:492–500. doi:10.1111/1469-0691.12144. 23398633

[22] Bhatti MM, Boonlayangoor S, Beavis KG, Tesic V. 2014. Evaluation of FilmArray and Verigene systems for rapid identification of positive blood cultures. J Clin Microbiol 52:3433–3436. doi:10.1128/JCM.01417-14. 25031445 PMC4313169

[23] Samuel LP, Tibbetts RJ, Agotesku A, Fey M, Hensley R, Meier FA. 2013. Evaluation of a microarray-based assay for rapid identification of gram-positive organisms and resistance markers in positive blood cultures. J Clin Microbiol 51:1188–1192. doi:10.1128/JCM.02982-12. 23363838 PMC3666768

[24] Sullivan KV, Turner NN, Roundtree SS, Young S, Brock-Haag CA, Lacey D, Abuzaid S, Blecker-Shelly DL, Doern CD. 2013. Rapid detection of gram-positive organisms by use of the Verigene gram-positive blood culture nucleic acid test and the BacT/Alert pediatric FAN system in a multicenter pediatric evaluation. J Clin Microbiol 51:3579–3584. doi:10.1128/JCM.01224-13. 23966484 PMC3889736

[25] Mancini N, Infurnari L, Ghidoli N, Valzano G, Clementi N, Burioni R, Clementi M. 2014. Potential impact of a microarray-based nucleic acid assay for rapid detection of gram-negative bacteria and resistance markers in positive blood cultures. J Clin Microbiol 52:1242–1245. doi:10.1128/JCM.00142-14. 24478405 PMC3993469

[26] Tojo M, Fujita T, Ainoda Y, Nagamatsu M, Hayakawa K, Mezaki K, Sakurai A, Masui Y, Yazaki H, Takahashi H, Miyoshi-Akiyama T, Totsuka K, Kirikae T, Ohmagari N. 2014. Evaluation of an automated rapid diagnostic assay for detection of gram-negative bacteria and their drug-resistance genes in positive blood cultures. PLoS One 9:e94064. doi:10.1371/journal.pone.0094064. 24705449 PMC3976387

[27] Kim JS, Kang GE, Kim HS, Kim HS, Song W, Lee KM. 2016. Evaluation of Verigene blood culture test systems for rapid identification of positive blood cultures. Biomed Res Int 2016:1081536. doi:10.1155/2016/1081536. 26904669 PMC4745370

[28] Martinez RM, Bauerle ER, Fang FC, Butler-Wu SM. 2014. Evaluation of three rapid diagnostic methods for direct identification of microorganisms in positive blood cultures. J Clin Microbiol 52:2521–2529. doi:10.1128/JCM.00529-14. 24808235 PMC4097746

[29] Ledeboer NA, Lopansri BK, Dhiman N, Cavagnolo R, Carroll KC, Granato P, Thomson R, Jr, Butler-Wu SM, Berger H, Samuel L, Pancholi P, Swyers L, Hansen GT, Tran NK, Polage CR, Thomson KS, Hanson ND, Winegar R, Buchan BW. 2015. Identification of gram-negative bacteria and genetic resistance eterminants from positive blood culture broths by use of the Verigene gram-negative blood culture multiplex microarray-based molecular assay. J Clin Microbiol 53:2460–2472. doi:10.1128/JCM.00581-15. 25994165 PMC4508435

[30] Siu GK, Chen JH, Ng TK, Lee RA, Fung KS, To SW, Wong BK, Cheung S, Wong IW, Tam MM, Lee SS, Yam WC. 2015. Performance evaluation of the Verigene gram-positive and gram-negative blood culture test for direct identification of bacteria and their resistance determinants from positive blood cultures in Hong Kong. PLoS One 10:e0139728. doi:10.1371/journal.pone.0139728. 26431434 PMC4592242

[31] Han E, Park DJ, Kim Y, Yu JK, Park KG, Park YJ. 2015. Rapid detection of gram-negative bacteria and their drug resistance genes from positive blood cultures using an automated microarray assay. Diagn Microbiol Infect Dis 81:153–157. doi:10.1016/j.diagmicrobio.2014.10.009. 25591999

[32] Zadka H, Raykhshtat E, Uralev B, Bishouty N, Weiss-Meilik A, Adler A. 2019. The implementation of rapid microbial identification via MALDI-ToF reduces mortality in gram-negative but not gram-positive bacteremia. Eur J Clin Microbiol Infect Dis 38:2053–2059. doi:10.1007/s10096-019-03640-w. 31359256

[33] Nielsen SL. 2015. The incidence and prognosis of patients with bacteremia. Dan Med J 62:B5128. 26183054

[34] Goto M, Al-Hasan MN. 2013. Overall burden of bloodstream infection and nosocomial bloodstream infection in North America and Europe. Clin Microbiol Infect 19:501–509. doi:10.1111/1469-0691.12195. 23473333

[35] CLSI. 2020. Performance standards for antimicrobial susceptibility testing, 30th ed. CLSI supplement M100. Clinical and Laboratory Standards Institute, Wayne, PA.

