# Supplementary material for: Identification and Antibiotic Susceptibility Patterns of Clinical Blood Culture Isolates Not Identified by a Rapid Microarray Diagnostic System
Source: Microbiol Spectr. 2021 Jun 30;9(1):10.1128/spectrum.00175-21. doi: 10.1128/spectrum.00175-21 (PMC8552754; doi:10.1128/spectrum.00175-21)
Supplement: SUPPLEMENTAL FILE 1 — Tables S1 and S2 and Fig. S1 to S4. Download SPECTRUM00175-21_Supp_1_seq3.pdf, PDF file, 0.5 MB [file spectrum00175-21_supp_1_seq3.pdf]

Table S1. Genera and Species Identified by the VERIGENE® System

Gram-Positive Blood Culture Test (BC-GP)	Gram-Negative Blood Culture Test (BC-GN)
Enterococcus faecalis	*Acinetobacter* spp.
Enterococcus faecium	*Citrobacter* spp.
Listeria spp.	*Enterobacter* spp.
Staphylococcus spp.	*Escherichia coli*
Staphylococcus aureus	*Klebsiella oxytoca*
Staphylococcus epidermidis	*Klebsiella pneumoniae*
Staphylococcus lugdunensis	*Proteus* spp.
Streptococcus spp.	*Pseudomonas aeruginosa*
Streptococcus agalactiae	
Streptococcus anginosus group	
Streptococcus pneumoniae	
Streptococcus pyogenes	

Table S2. Overview of Antimicrobial Susceptibility Testing in this Study	
**Gram positive organisms**	**Gram negative organisms**
**Antimicrobial Susceptibilities Routinely Tested**	
Ampicillin	Amikacin
Clindamycin	Amoxicillin/Clavulanate
Cefazolin	Ampicillin
Doxycycline	Aztreonam
Daptomycin	Cefazolin
Erythromycin	Cefepime
Nitrofurantoin	Ceftazidime
Cefoxitin	Ceftriaxone
Gentamicin	Ciprofloxacin
Levofloxacin	Ertapenem
Linezolid	Gentamicin
Moxifloxacin	Imipenem
Oxacillin	Levofloxacin
Penicillin	Meropenem
Rifampin	Nitrofurantoin
Trimethoprim/Sulfamethoxazole	Piperacillin/Tazobactam
Quinupristin/Dalfopristin	Tetracycline
Tetracycline	Tobramycin
Vancomycin	Trimethoprim/Sulfamethoxazole
**Species for Which Antimicrobial Susceptibilities Were not Routinely Tested[1]**	
Aerococcus species	*Campylobacter* species
Bacillus species	*Haemophilus influenzae*
Dermacoccus nishinomiyaensis	*Neisseria* species
Gemella species	*Pasteurella multocida*
Lactobacillus species	*Rhizobium radiobacter*
Leuconostoc species	*Roseomonas mucosa*
Micrococcus species	Obligate anaerobes
Rothia species	
Streptococcus-nutritionally deficient	
Obligate anaerobes	

[1]Species listed here had at least 1 isolate for which routine antimicrobial susceptibility testing was not performed

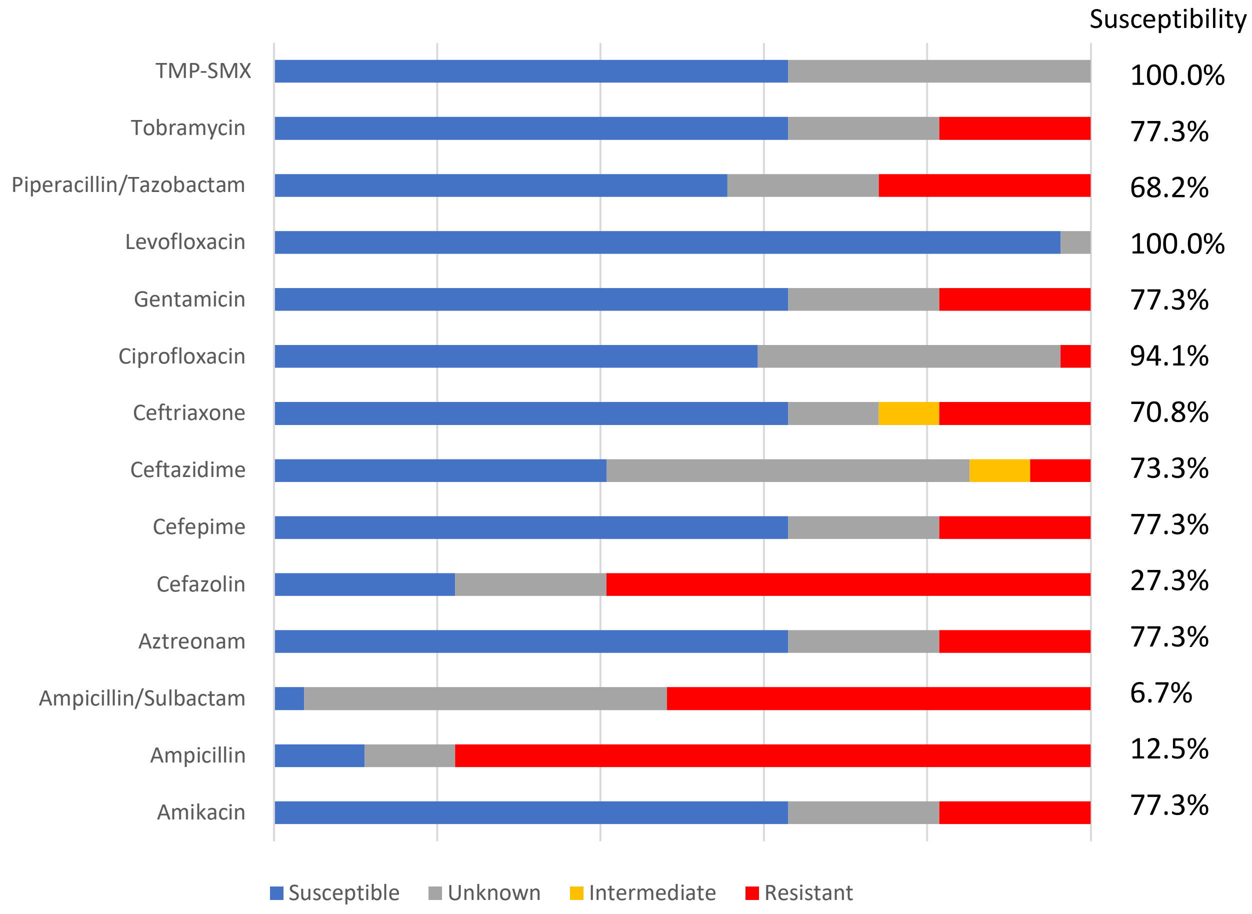

Figure S1. Resistance Patterns for Aerobic GNRs Blood Culture Isolates Collected in an ICU but Not Identified by VERIGENE®. Composite data from antibiotic susceptibility testing along with intrinsic resistance shown. Isolates were presumed to be susceptible if susceptibility testing was not done on that isolate but was done on a separate isolate of the same species from the same patient during the same admission. Percent susceptibility displayed reflects the percentage of susceptible isolates among all isolates with either antimicrobial susceptibility testing data or known intrinsic resistance. Percent susceptibility is not displayed in cases where more than 50% of isolates were not tested for susceptibility to a given antibiotic. N= 27.

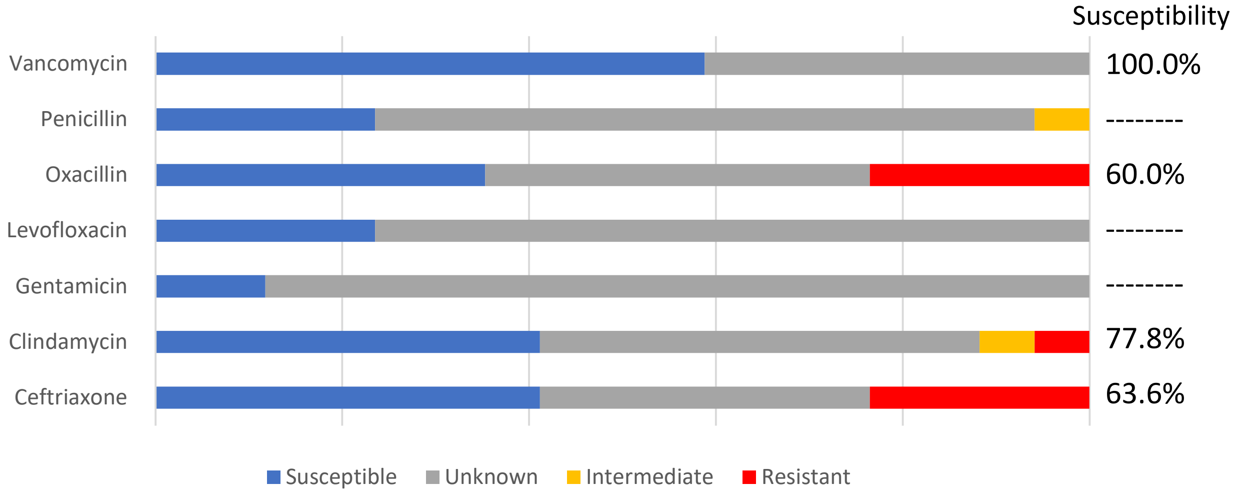

Figure S2. Resistance Patterns for Aerobic GPCs Blood Culture Isolates Collected in an ICU but Not Identified by VERIGENE®. Composite data from antibiotic susceptibility testing along with intrinsic resistance shown. Isolates were presumed to be susceptible if susceptibility testing was not done on that isolate but was done on a separate isolate of the same species from the same patient during the same admission. Percent susceptibility displayed reflects the percentage of susceptible isolates among all isolates with either antimicrobial susceptibility testing data or known intrinsic resistance. Percent susceptibility is not displayed in cases where more than 50% of isolates were not tested for susceptibility to a given antibiotic. N= 17.

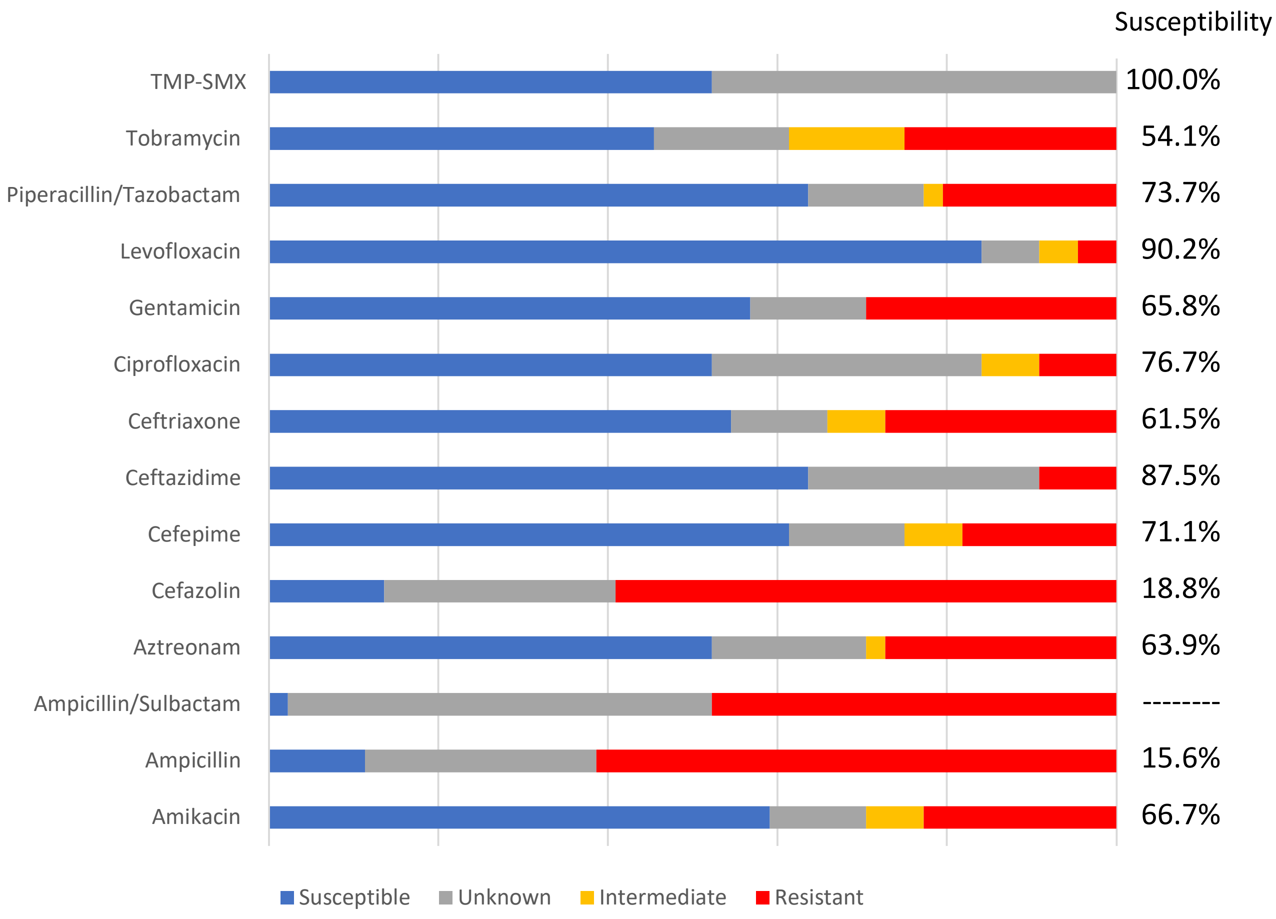

Figure S3. Resistance Patterns for Aerobic GNRs Blood Culture Isolates Collected Outside of an ICU and Not Identified by VERIGENE®. Composite data from antibiotic susceptibility testing along with intrinsic resistance shown. Isolates were presumed to be susceptible if susceptibility testing was not done on that isolate but was done on a separate isolate of the same species from the same patient during the same admission. Percent susceptibility displayed reflects the percentage of susceptible isolates among all isolates with either antimicrobial susceptibility testing data or known intrinsic resistance. Percent susceptibility is not displayed in cases where more than 50% of isolates were not tested for susceptibility to a given antibiotic. N= 44.

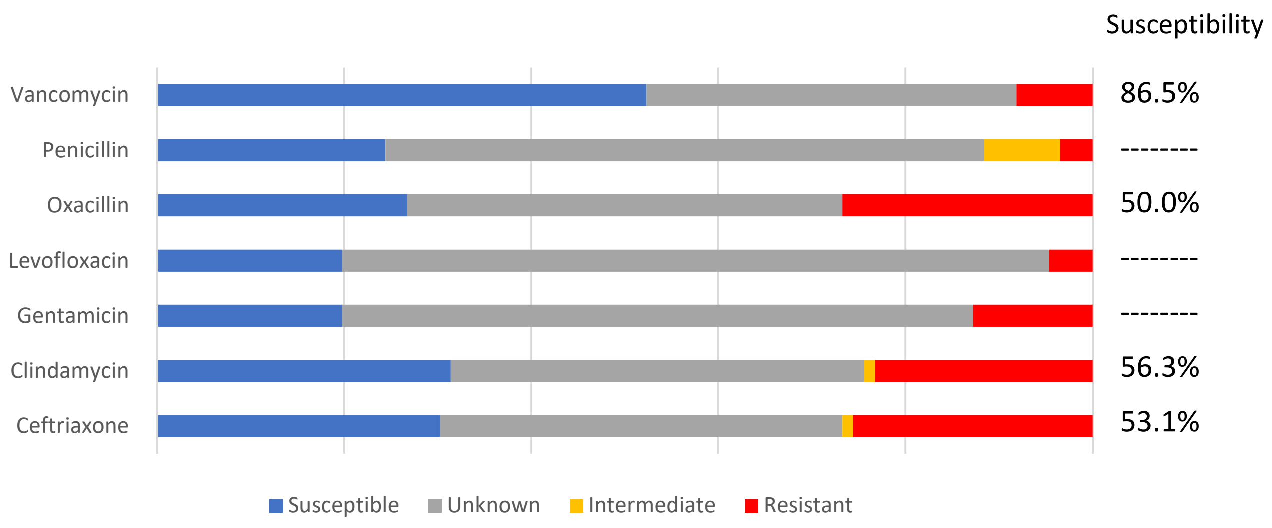

Figure S4. Resistance Patterns for Aerobic GPCs Blood Culture Isolates Collected Outside of an ICU and Not Identified by VERIGENE®. Composite data from antibiotic susceptibility testing along with intrinsic resistance shown. Isolates were presumed to be susceptible if susceptibility testing was not done on that isolate but was done on a separate isolate of the same species from the same patient during the same admission. Percent susceptibility displayed reflects the percentage of susceptible isolates among all isolates with either antimicrobial susceptibility testing data or known intrinsic resistance. Percent susceptibility is not displayed in cases where more than 50% of isolates were not tested for susceptibility to a given antibiotic. N= 86.
